# Cervicomedullary hemangioblastoma treated with bevacizumab

**DOI:** 10.1093/noajnl/vdaa076

**Published:** 2020-09-03

**Authors:** Gloria Mak, Almunder Algird, Jeffrey Greenspoon, John Provias, Hal Hirte

**Affiliations:** 1 Department of Medicine, McMaster University, Hamilton, Ontario, Canada; 2 Department of Surgery, McMaster University, Hamilton, Ontario, Canada; 3 Department of Oncology, McMaster University, Hamilton, Ontario, Canada; 4 Department of Pathology and Molecular Medicine, McMaster University, Hamilton, Ontario, Canada

**Keywords:** bevacizumab, hemangioblastoma

Hemangioblastomas are WHO grade I vascularized tumors that primarily occur sporadically. They are commonly found below the tentorium, with 5% located in the brainstem.^1^ Hemangioblastomas are composed of stromal cells, which highly express vascular endothelial growth factor (VEGF), and are surrounded by a capillary network, composed of endothelial cells expressing VEGF receptor.^1^ We describe a case where bevacizumab, a humanized monoclonal antibody targeting VEGF, was successful in treating a surgical and stereotactic radiosurgical refractory hemangioblastoma.

## Case Report

**Figure 1.**
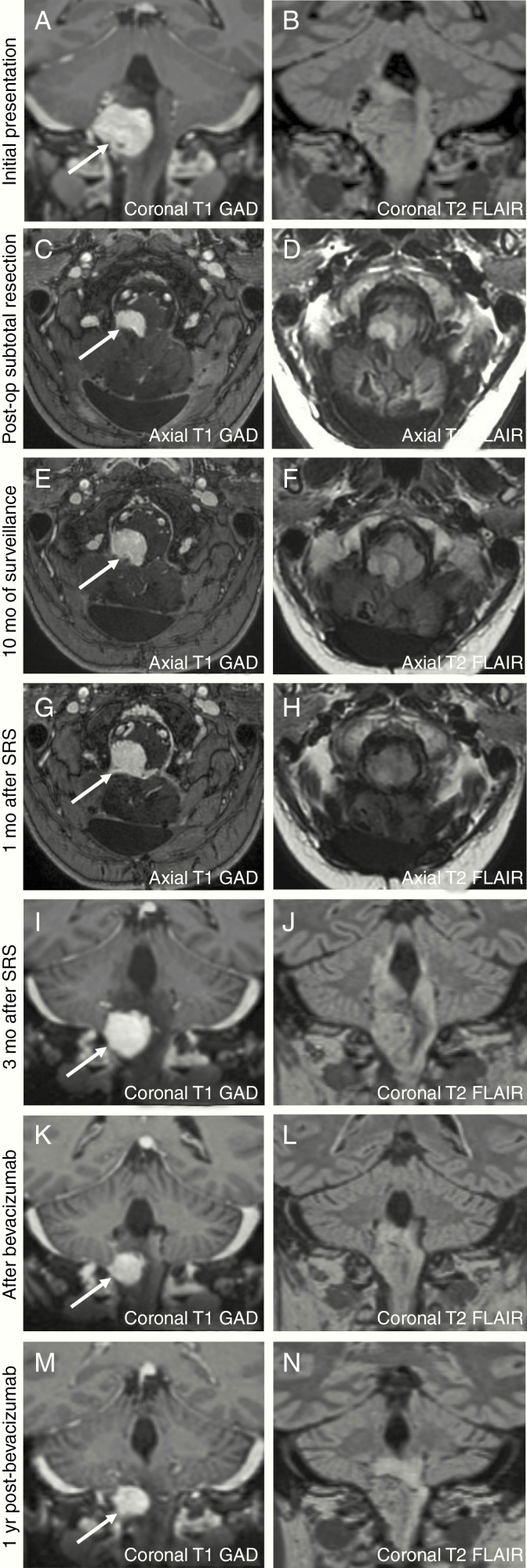
At the initial presentation, MRI revealed a mass at the cervicomedullary junction measuring 2 × 2.5 × 2.3 cm (A and B). After subtotal surgical resection (C and D), the size of the hemangioblastoma decreased to 1.6 × 0.6 × 1.1 cm. With 10 months of surveillance, the hemangioblastoma increased to 1.6 × 1.0 × 1.4 cm (E and F). There was an enlargement of the hemangioblastoma 1-month (mo) post-stereotactic radiosurgery, measuring 1.9 × 1.5 × 1.8 cm, with associated edema and hydrocephalus (G and H). Ongoing surveillance revealed a further increase of the hemangioblastoma (2.0 × 1.8 × 2.0 cm) and increased surrounding edema (I and J). After bevacizumab therapy, there was a decrease in the size of the hemangioblastoma, measuring 1.4 × 1.5 × 1.7 cm (K and L) and reduction of surrounding edema. Over 1 year of clinical surveillance, the hemangioblastoma has remained stable in size, measuring 1.4 × 1.6 × 1.7 cm (M and N).

A 41-year-old immunocompetent female with no significant comorbidities presented with a 1-year history of progressive nausea and vomiting. Investigations for a gastrointestinal cause of her nausea and vomiting were unremarkable. She subsequently developed right-sided numbness that prompted a MRI brain to be performed. The MRI demonstrated a homogenous enhancing intra-axial mass located at the right dorsolateral cervicomedullary junction, measuring 2 × 2.5 × 2.3 cm (Figure 1A and B).

She underwent a suboccipital craniotomy for intended total resection, however, attempts triggered multiple episodes of bradycardia, and therefore only a biopsy was performed. Unfortunately, the pathology was non-diagnostic. As such, she was managed with clinical and imaging surveillance.

Over the next 3 months, she continued to experience nausea, vomiting, right-sided numbness with progressive headaches, and right-sided weakness with ataxia. Repeat imaging showed an increased size of the mass (2.3 × 3 × 2.7 cm) and hydrocephalus.

A second attempt to totally resect the mass again triggered episodes of bradycardia and a transient episode of asystole. Instead, a subtotal resection was achieved. Postoperatively, the mass measured 1.6 × 0.6 × 1.1 cm (Figure 1C and D).

## Pathology

**Figure 2.**
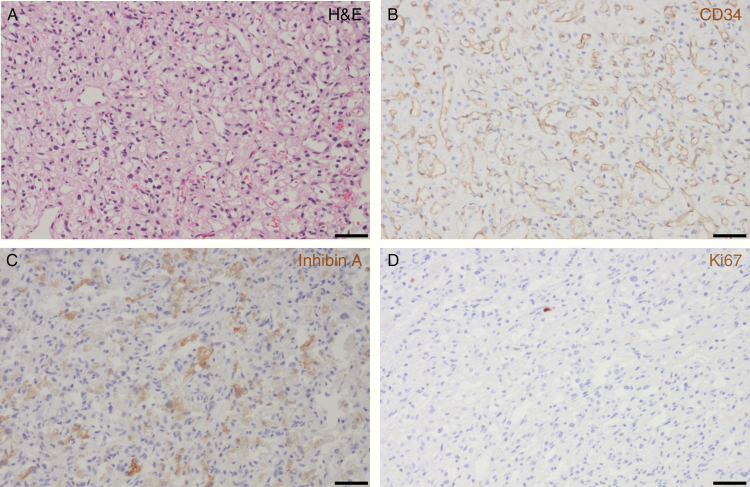
Surgical resection from the cervicomedullary mass shows typical features of hemangioblastoma with numerous irregular small capillary-type vascular channels and an intervening cell population (so-called “stromal” cells) on hematoxylin and eosin (H&E) staining, 200× magnification (A) with the scale bar measuring 50 µm. There are prominent capillary-type vascular spaces highlighted by CD34-positive immunohistochemistry staining for endothelial cells, 200× magnification (B) with the scale bar measuring 50 µm. The non-vascular tumor cells (“stromal” cells) exhibit characteristic inhibin A positivity by immunohistochemistry, 200× magnification (C) with the scale bar measuring 50 µm. There is a very low proliferative activity of the tumor with only a rare tumor cell nucleus positive for Ki67 on immunohistochemistry, 200× magnification (D) with the scale bar measuring 50 µm.

Histologic examination of the subtotal resected mass from the cervicomedullary region demonstrated a tumor composed of stromal cells with clear, vacuolated cytoplasm and an intervening capillary network (Figure 2A). The vascular network was comprised of endothelial cells, as they were positive for CD34 (Figure 2B). The stromal cells of the resected specimen demonstrated a characteristic expression of inhibin A (Figure 2C). Additionally, the tumor itself had a low proliferative index, as demonstrated by scant Ki67-positive immunohistochemistry (Figure 2D). As such, the histopathological features of the partially resected mass were consistent with a WHO grade I hemangioblastoma.

## Subsequent Clinical Course

Clinical and radiographical surveillance for 10 months after the subtotal resection revealed that the hemangioblastoma increased to 1.6 × 1.0 × 1.4 cm (Figure 1E and F). Due to unsuccessful attempts of surgical resection, the patient was instead placed on dexamethasone and underwent stereotactic radiosurgery (SRS) to the right dorsolateral brainstem at a dose of 2100 cGy in 3 fractions. She was subsequently weaned off dexamethasone, however within 1 month developed worsening nausea, vomiting, weakness, ataxia, and was no longer able to ambulate. An urgent MRI showed an interval increase in the size of the hemangioblastoma (1.9 × 1.5 × 1.8 cm), acute hydrocephalus, and adjacent edema (Figure 1G and H). She was placed on dexamethasone 4 mg twice daily and admitted to hospital for placement of a ventriculoperitoneal shunt.

Three months later, she developed dysphagia and required placement of a PEG tube. Repeat imaging showed both an increase in size of the hemangioblastoma (2.0 × 1.8 × 2.0 cm) and surrounding edema (Figure 1I and J). Her functional status declined to a Karnofsky Performance Score (KPS) of 20.

A discussion regarding the use of bevacizumab to treat her hemangioblastoma was initiated. A dose of 10 mg/kg every 2 weeks was administered for 4 months. Bevacizumab therapy was stopped due to the patient’s financial constraints. During the bevacizumab treatment, her dysphagia improved, such that she no longer required the PEG tube, she regained her ability to ambulate and was weaned to dexamethasone 0.5 mg daily. There was also radiographical improvement of the size of the hemangioblastoma (1.4 × 1.5 × 1.7 cm) and reduction of surrounding edema (Figure 1K and L).

She has been followed clinically with repeat MRI every 3 months for 1 year off of dexamethasone. Her KPS improved to 60 and the size of the hemangioblastoma has remained stable (1.4 × 1.6 × 1.7 cm) (Figure 1M and N).

## Discussion

Hemangioblastomas are highly vascularized tumors that are commonly found below the tentorium. The majority of hemangioblastomas occur sporadically (~70%), while the remaining occurs in the context of the autosomal dominant von Hippel–Lindau (VHL) disease (~30%).^1^ Histologically, hemangioblastomas are comprised of stromal cells that are surrounded by a capillary network. Stromal cells express hypoxia-inducible factor (HIF) 1A, HIF2A, and VEGF, while the endothelial cells of the capillary network express the VEGF receptor.^2^ In both sporadic and inherited etiologies of hemangioblastomas, the VHL–HIF pathway is thought to play a role in the pathogenesis of this highly vascular tumor.^2^ VHL is a tumor-suppressor gene that encodes for the VHL protein, which is part of a ubiquitin ligase protein complex that regulates the proteolytic degradation of HIF1A and HIF2A by binding to their alpha subunits.^2^ In the absence, or inactivation, of VHL protein, there is HIF1A and HIF2A activation of downstream target genes, which includes VEGF, thus promoting angiogenesis.^2^ Hemangioblastomas have also been found to express Delta-like 4/Notch, CXC motif chemokine receptor 4/stromal cell-derived factor 1 alpha, and EphrinB2/Ephrin type B receptor 4 angiogenic signaling pathways.^3^ The interplay between these angiogenic signaling pathways is thought to contribute to the high vascularity of hemangioblastomas.

The treatment of choice for symptomatic hemangioblastomas is complete surgical resection; however, medullary hemangioblastomas can be surgically challenging and lead to morbidity and mortality.^4^ In circumstances where intracranial hemangioblastomas are not amenable to surgical resection, SRS has demonstrated significant efficacy long term.^5^ However, complications of SRS can include hydrocephalus, peritumor edema, and radiation necrosis.

Bevacizumab is a humanized monoclonal antibody that targets VEGF to prevent it from binding to the VEGF receptor. As such, bevacizumab inhibits tumor angiogenesis by preventing the proliferation and migration of vascular endothelial cells. There are a few case reports that have utilized bevacizumab therapy for single and multiple hemangioblastomas not amenable to surgical resection.^6–8^ However, in certain instances, clinical and radiographical progression was observed.^7,8^ Our case highlights the efficacy of limited bevacizumab therapy for intracranial hemangioblastomas unsuccessfully treated with surgery and radiosurgery.

Specific to our clinical case, it could be argued that the patient’s deterioration in clinical status post-SRS was secondary to pseudo-progression from the adverse effects of radiation, versus an increase in the size of the hemangioblastoma. Peritumor edema and radiation necrosis from SRS are thought to arise from a number of mechanisms, including disruption of the blood–brain barrier, radiation-induced damage to microglia and astrocytes, release of inflammatory cytokines, and upregulation of angiogenesis factors, such as VEGF and hypoxia-related factors, which increase vascular permeability resulting in extracellular edema and radiation necrosis.^9,10^ Dexamethasone is thought to dampen peritumor edema by indirect inhibition of VEGF,^10^ but despite the patient being on dexamethasone, her clinical symptoms continued to progress. Radiographically, there was an increase in the size of the hemangioblastoma, which can be distinguished from pseudo-progression by comparing T1-weighted gadolinium-enhanced images with T2-weighted images. Congruency between the T1-weighted gadolinium-enhanced images and the hypointense tumor margin on T2-weighted images indicates a “T1/T2 match,” and any associated increase in tumor size is associated with tumor progression. The lack of clear tumor margins on T2-weighted images compared to the T1-weighted gadolinium-enhanced images indicates a “T1/T2 mismatch,” and therefore an increase in tumor size is associated with pseudo-progression secondary to radiation effects.^11^ In our clinical case, there appears to be an adequate T1/T2 match, suggesting that tumor growth contributed to the patient’s decline versus pseudo-progression from radiation-induced edema.

Pursuant to the patient’s clinical improvement with bevacizumab, it is possible that the benefit was not only from the reduction in the size of the hemangioblastoma, but also from treating the surrounding dexamethasone-refractory peritumor inflammatory changes from SRS. Given that surgical histology was not obtained post-SRS, it would be difficult to tease apart the exact mechanism by which bevacizumab improved the patient’s clinical status. Both reactive astrocytes and endothelial cells within peri-radiation-induced angiogenic regions have been shown to abundantly express VEGF.^12^ As such, it is possible that radiation-induced inflammatory changes contributed to the growth of the patient’s hemangioblastoma via upregulation of the VEGF/VEGF receptor angiogenic signaling pathway within and surrounding the tumor itself. Moreover, bevacizumab has been previously demonstrated to improve dexamethasone-refractory radiation-induced peritumor edema and necrosis.^12,13^ Therefore, the administration of bevacizumab may have inhibited the radiation-induced upregulation of VEGF, which led to a decrease in tumor size, improvement of the surrounding peritumor edema, and ultimately contributed to the improvement of the patient’s clinical status.

This clinical case highlights how inhibition of VEGF, through the use of bevacizumab, can provide long-term clinical and radiographic improvement of cervicomedullary hemangioblastomas that are refractory to surgical and radiosurgical intervention. Moreover, in instances where hemangioblastomas are refractory to surgical and radiosurgical intervention, bevacizumab may have a multifaceted role, where it not only inhibits VEGF signaling intrinsic to the hemangioblastoma, but also dampens VEGF signaling induced by inflammatory changes that arise post-SRS which can contribute to further tumor progression and adverse radiation effects.

## Notes

**Authorship Statement**. G.M. was involved in the acquisition and interpretation of data, drafting of the manuscript, and patient care. A.A. was involved in the acquisition and interpretation of data, critical revision of the manuscript, and patient care. J.G. was involved in the acquisition and interpretation of data, critical revision of the manuscript, and patient care. J.P. was involved in the acquisition and interpretation of data. H.H. was involved in the acquisition and interpretation of data, critical revision of the manuscript, and patient care.
